# Isolation and sequencing of the spoilage yeast *Kregervanrija delftensis*

**DOI:** 10.1128/MRA.00397-23

**Published:** 2023-09-15

**Authors:** Joseph H. Collins, Amparo Cosio, Garrett Brogan, Lucas Chico, Alexandra Rozen, Ellen Thomson, Eric M. Young

**Affiliations:** 1 Department of Chemical Engineering, Worcester Polytechnic Institute, Worcester, Massachusetts, USA; University of California, Riverside, California, USA

**Keywords:** craft cider, cultured products, yeast genomics, nanopore sequencing

## Abstract

New sequencing workflows can enable the genomics of microbes isolated from craft beverages. Here, we use hybrid, short, and long-read sequencing to assemble the genome of a yeast isolated from cider, *Kregervanrija delftensis* NCC-J. The *Kregervanrija* genus has few genomes available; thus, this contributes to yeast genomics.

## ANNOUNCEMENT

Unintended yeasts in fermented beverages can lead to spoilage, inconsistency, and unique flavor profiles (1, 2). Targeted internal transcribed spacer (ITS) sequencing can consistently identify the genus of the yeast but not the species (3). Large-scale Whole Genome Sequencing (WGS) of beer yeasts can interrogate isolates, guide selection and breeding of superior strains, or select genome engineering targets (4). Thus, we reasoned that low-cost *de novo* WGS can be applied directly after ITS sequencing. Therefore, we applied our Prymetime (5) pipeline to a yeast isolated from cider. A strain designated NCC-J was isolated from North Country Hard Cider’s (Rollinsford, NH) Bitter Brothers Blend process, which produces a hard cider with a strong tart flavor. Initial ITS sequencing placed NCC-J as *Kregervanrija delftensis*. It was then sequenced with both nanopore and Illumina technologies, and the resulting reads were assembled using the Prymetime pipeline, which is a double hybrid assembly pipeline using both short and long reads and linear and circular assemblers (5). The resulting genome sequence (GenBank accession GCA_019155655.1) is the highest quality assembly within *Kregervanrija* (see Table 1). This demonstrates that WGS can be used much earlier in characterization of fermentations and contributes to the genomics of a nonconventional yeast genus.

**TABLE 1:** Genome assembly, annotation, and completeness statistics for *Kregervanrija* genomes^*a*^

Strain	*Kregervanrija fluxuum* NRRL YB-4273	*K. delftensis* NRRL Y-7119	*K. delftensis* NCC-J
Number of contigs	1,789	1,236	20
Contig N50 (Mbp)	0.19	0.22	1.10
Largest contig (Mbp)	0.55	0.54	2.24
Total assembly length (Mbp)	12.51	12.31	12.74
GC (%)	31.78	30.46	30.68

^*a*^ Both *K. delftensis* NRRL Y-7119 and *K. fluxuum* NRRL YB-4273 (6) were obtained from Shen et al*.*(6). *K. delftensis* NCC-J is from this work.

To isolate yeast from the cider process, a 1 mL sample of cider was plated onto YPD agar (10 g/L yeast extract, 20 g/L peptone, 20 g/L glucose, and 15 g/L agar) and incubated for 2 d at 30°C. Colonies were picked and cultivated overnight in YPD at 30°C. Colony NCC-J was identified as *K. delftensis* by ITS sequencing using primers defined by Kurtzman (3). Genomic DNA from *K. delftensis* NCC-J was isolated using Promega’s Genomic DNA Isolation Kit (Promega, A1120) and prepared for sequencing on the Illumina iSeq using the Nextera DNA Flex Library Prep Kit (Illumina, 20018704) along with the Nextera DNA CD Indexes (Illumina, 20018707), and the nanopore DNA library was prepared using the Rapid Barcoding Kit (Oxford Nanopore Technologies, SQK-RBK004).

For each software step, the default parameters were used unless otherwise noted. Nanopore fast5 files were basecalled using Guppy v2.3.5 (Oxford Nanopore base caller, FAST model). The subsequent fastq files were demultiplexed using the EPI2ME interface (Metrichor, Oxford, UK). Illumina reads were demultiplexed using the native software on the iSeq 100 machine. This resulted in 83,300 Nanopore reads with an average length of 15,995 bp (104.6X genome coverage) and 1,020,923 paired-end Illumina reads with an average length of 148 bp (23.7X genome coverage) after basecalling and demultiplexing, which also filtered out low-quality reads according to default parameters. Using these reads, the *K. delftensis* NCC-J genome was assembled using the Prymetime v0.2 pipeline, which uses both flye and Unicycler as the underlying assemblers (5).

The NCC-J genome assembly was compared to two other publicly available assemblies in the *Kregervanrija* genus: *K. delftensis* NRRL Y-7119 assembly GCA_003707205.2 and *K. fluxuum* NRRL YB-4273 assembly GCA_003707275.2 (6). Assembly statistics were generated using QUAST v4.6.3 (7). The results are reported in Table 1. The *K. delftensis* NCC-J assembly has greater contiguity as measured by number of contigs, contig N50, and the largest contig.

## Data Availability

Data were deposited at GenBank under BioProject PRJNA706002, BioSample SAMN18109229, SRA numbers SRR13883851 and SRR13883852, and assembly number GCA_019155655.1.
